# Fast detection of protein kinase B in chrysin treated colorectal cancer cells using a novel multicore microfiber biosensor

**DOI:** 10.1038/s44172-024-00332-y

**Published:** 2024-12-26

**Authors:** Zhen Tian, Hongzhuan Xuan, Yicun Yao, Shengyu Hao, Zhichao Zhang, Bingyuan Zhang, Jingao Zhang, Liqiang Zhang, Xinzhu Sang, Jinhui Yuan, Gerald Farrell, Qiang Wu

**Affiliations:** 1 State Key Laboratory of Information Photonics and Optical Communications, Beijing University of Posts and Telecommunications, Beijing, China; 2 School of Physics Sciences and Information Technology, Liaocheng University, Liaocheng, China; 3 School of Life Science, Liaocheng University, Liaocheng, China; 4 Photonics Research Centre, School of Electrical and Electronic Engineering, City Campus, Technological University Dublin, Dublin, Ireland; 5 Faculty of Engineering and Environment, Northumbria University, Newcastle Upon Tyne, United Kingdom; 6 Key Laboratory of Optoelectronic Information Science and Technology of Jiangxi Province, Nanchang Hangkong University, Nanchang, China

**Keywords:** Optical sensors, Biomedical engineering, Fibre optics and optical communications

## Abstract

Rapid and accurate determination of target proteins in cells provide essential diagnostic information for early detection of diseases, evaluation of drug responses, and the study of pathophysiological mechanisms. Traditional Western blotting method has been used for the determination, but it is complex, time-consuming, and semi-quantitative. Here, a tapered seven-core fiber (TSCF) biosensor was designed and fabricated. By immobilizing protein kinase B (PKB), also known as AKT, antibody onto TSCF surface, the microfiber biosensor can be used for quantitatively detecting the AKT level in solution concentrations as low as 0.26 ng/mL. To test the reliability of the TSCF sensing method in a medical application, the TSCF biosensor was used to study the relationship between chrysin’s anticancer effect and the concentration of AKT in a human colorectal cancer cell line (LoVo cells). The results reveal that the inhibitory effect of chrysin on LoVo cells is positively correlated with the dose, agreeing well with the equivalent results using the traditional Western blotting method.

## Abstract (web summary)

Zhen Tian, Hongzhuan Xuan, and colleagues show an optical fiber sensor for measuring concentration changes of protein kinase B, also known as AKT, extracted from human Lovo cells. Their biosensor allows studying anti-cancer mechanisms in the human body.

## Introduction

Proteomics is the science which studies protein compositions and protein changes in cells, tissues or organisms. It is mainly used for early detection of disease, evaluation of treatment responses and research on pathophysiological mechanisms^1^. The serine/threonine protein kinase B (PKB), also known as AKT, is one type of cellular protein. It is a central node of the targeted PI3K signaling pathway and important in regulating cell survival, tumor formation, angiogenesis, insulin signaling, and growth factor-induced neuronal survival^2,3^. Aberrant activation of AKT has been shown to occur frequently in human cancers^4,5^, including colorectal cancer^6^, liver cancer, etc.^7,8^ Currently, AKT has been the subject of intense research into PI3 kinase transduction signaling, and has become a hot target for cancer treatment, stroke treatment and diabetes drugs^7–9^. Generally, AKT has three ubiquitously expressed isoforms as AKT_1_, AKT_2_, and AKT_3_ that mediate a number of downstream events controlled by PI3-kinase^10^. They are activated when their residues, of Ser-473 and Thr-308, within the P-loop of the protein kinase domain are phosphorylated and referred to as p-AKT^7,11^. To study how AKT is involved in physiological processes, it is necessary to examine the AKT and p-AKT level, and the relative changes between them. At present, the main AKT and p-AKT detection technologies include semi-quantitative western blotting (WB) and enzyme-linked immuno-absorbent assay (ELISA)^12,13^. In addition, high-resolution mass spectrometry is also available^14,15^. However, those methods have several shortcomings. For example, the process time of WB is lengthy, usually taking about 8–20 h. This is because of the complexity of the steps involved, which including electrophoresis, electro transfer, enzyme immunolocalization, washing, chemiluminescence, and gel image analysis, which could be easily extended to several days if there is a need to detect multiple target proteins^16,17^. In addition the need for multiple steps and the long duration, along with protein degradation, may make the process more prone to failure. Also, the ELISA needs additional secondary antibody incubation, which may result in unwanted cross-reactions^18,19^. In contrast, high-resolution mass spectrometry reveals all the proteins present in the sample, which is quantitative. However, it is much more expensive and the operator must be well-trained, which restricts its widespread use and application^10^.

Optical fiber sensors have been intensely researched and developed because of their unique advantages, such as compactness, resistance to corrosion and electromagnetic interference, applicability to harsh environments and so on^20,21^. They are typically used to measure a range of direct physical parameters, including temperature^22,23^, refractive index (RI)^24^, stress^25,26^, pressure^27^, and vibration^28^, etc. However, by immobilizing a specific capture or functional layer on an optical fiber sensor, the sensor can be utilized as a biosensor as an alternative to fluoroimmunoassay, radioimmunoassay, and enzyme-linked immunoassay^19,29^. Typically, capture layers work by altering their RI in response to the presence of a specific compound, with the change in RI in turn detected by the underlying optical fiber sensor structure. If the optical fiber biosensor surface is functionalized with the specific AKT antibody (AKT-Ab), the sensor can be used for simple and rapid quantitative detection of AKT levels in a solution.

Until now, several configurations of fiber optic biosensors have been developed such as unclad fiber^30^, U-shape fiber^31^, D-shape fiber^32^, tapered fiber^33,34^, end-face reflected fiber^35^, fiber gratings^36,37^, special optical fibers^38,39^, and a microfiber interferometer^40^, etc. Among them, a microfiber interferometer based on a tapered optical fiber has been widely studied due to its strong evanescent field, which ensures a very high level of interaction with the local environment and thus high sensor sensitivity. Tapered single mode-multimode-single mode (TSMS) fiber structures have attracted wide research interest and a comprehensive review of these structures can be found in ref. ^20^. In the TSMS fiber structure, the multi-mode fiber section can have different forms, such as no-core, small core, multicore, etc. For multicore fibers (MCFs), when the distance between each core is small enough, multiple supermodes will be generated, and interferences between different supermodes will occur. More importantly, multiple supermodes can be generated without meeting the non-adiabatic condition for a conventional tapered single mode fiber sensor structure. Moreover, the cladding region of an MCF is thin, so the evanescent field is strong and this leads to high sensitivity. As a result, the application of an MCF in optical sensing systems has attracted more and more attention due to its high sensitivity, simple fabrication process and good repeatability^41,42^.

Chrysin is a flavonoid found in many plants, especially propolis, which has many potential medicinal properties^43^. Studies have shown that chrysin has the functions of inhibiting tumor cell proliferation^44^, inducing tumor cell apoptosis^45^, inhibiting tumor angiogenesis^46^, reversing multidrug resistance of tumor cells^47^, and has become one of the most important compounds which are beneficial to human health. Among the various pharmacological effects displayed by chrysin, anticancer activity is the most promising one^43^. Specifically, chrysin inhibits the development and progression of many types of cancer cells present in hepatocellular carcinoma^8^, prostate^48^, skin^49^, and lung cancer^50^. The mechanism by which chrysin inhibits the activity of LoVo cells has been extensively studied^51,52^. However, the details of this mechanism still remain unclear, particularly due to the lack of fast detection method. It is very necessary to understand the underlying action mechanisms of chrysin to determine its effectiveness as a potential treatment agent.

In this work, a high sensitivity microfiber biosensor is demonstrated based on a structure of tapered seven-core fiber (TSCF). By functionalizing this microfiber biosensor with AKT-Ab, a number of AKT samples of 0.5, 5, 50, 500, and 5000 ng/mL were used for a sensor performance test, with the sensing limit found to be low as 0.26 ng/mL. Subsequently, to demonstrate the usefulness of the proposed sensor in an actual application, the microfiber biosensor was used to study the mechanism by which chrysin inhibits LoVo cell’s viability. This involves fast detection of concentration changes for the AKT and p-AKT extracted from LoVo cells treated with different doses of chrysin. The results can provide a theoretical and experimental basis for elucidating the anti-tumor mechanism of chrysin and broaden the application of chrysin in cancer treatment. The expression levels of the AKT and p-AKT proteins were also detected by a traditional WB method, and the results were compared with those obtained by the proposed microfiber biosensor.

## Methods

### Multicore fiber and its sensing performance simulation

**Fig. 1:**
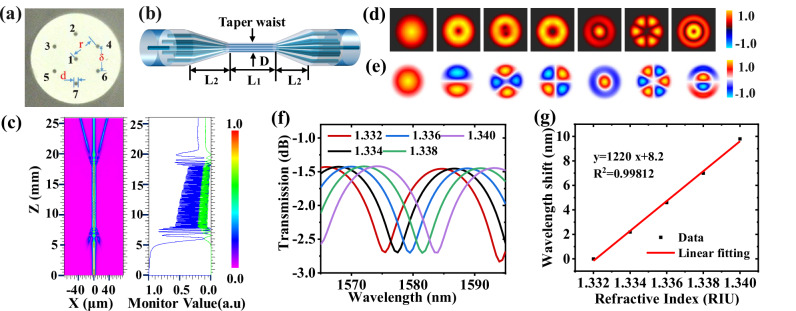
Schematic structure diagram of sensing fiber and its sensing performance simulation. **a** The cross-section structure of the SCF used. **b** Schematic diagram of the TSCF structure. **c** The simulated normalized power and propagation profile (blue curve for central core, green curve for one satellite core) along the TSCF at wavelength 1550 nm. **d** The simulated mode profiles of the fundamental and higher-order supermodes in the TSCF with *D* equal to 12 μm. **e** The corresponding simulated electric field component *Ex* of the seven supermodes. **f** The simulated transmission spectra. **g** The linear fitting results between the dip wavelength and surrounding RI over the RI range from 1.332 to 1.340. (SCF seven-core fiber, TSCF tapered seven-core fiber, RI refractive index).

The multicore fiber (MCF) used in the study is a commercially available product (SM-7C1500, Fibercore Company), whose cross-section structure is shown in Fig. 1a. Figure 1b shows the schematic diagram of the TSCF structure. In this work, the MCF was tapered adiabatically with an optical glass processing system (3SAE Technologies, USA). And the diameter profile of the TSCF is shown in Fig. S1 in Supplementary Material. The distances r and δ between the cores of the MCF affect the coupling between the core modes after the tapering process. Smaller values of r and δ will increase the overlap of the mode fields of different cores, resulting in a larger intermodal coupling coefficient, which in turn reduces the interference length and decreases the free spectral range of the interference spectrum^38,39,53^. The MCF used in this work has a distance of 35 μm between the cores^38,39^. Figure 1c shows the simulated normalized power and propagation profile (blue curve for central core, green curve for one satellite core) along the TSCF at wavelength 1550 nm. Figure 1d shows the simulated mode profiles of the fundamental and higher-order supermodes in the TSCF with *D* equal to 12 μm. and Fig. 1e is the corresponding simulated electric field component *Ex* of the seven supermodes. Figure 1f, g show the simulated transmission spectra through the central core and its linear fitting result between the dip wavelength and surrounding RI in the RI range from 1.332 to 1.340. As seen from Fig. 1g, an RI sensitivity of 1220 nm/RIU can be achieved within RI range from 1.332 to 1.340. (Supplementary Material 1 contains a more thorough description).

### Experimental setup and its sensing performances

**Fig. 2:**
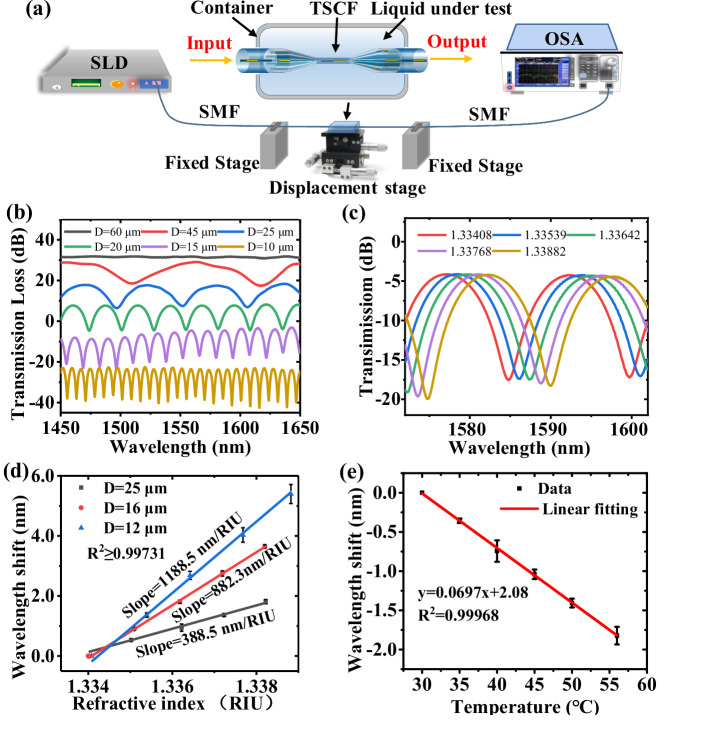
Schematic diagram of experimental setup and the sensing performances of TSCF. **a** A schematic diagram of experimental setup used for the TSCF sensing. **b** Spectral responses of the TSCF for different values of *D*. **c** The transmission curves of the TSCF with tapered diameter *D* of 12 μm when the RI of the solutions varies around a value of ~1.333. **d** The RI sensitivity of the TSCF around an RI value of 1.333. The blue, red, and black curves are the linear fits of the dip wavelength shift for *D* of 12, 16, and 25 μm, respectively. **e** The temperature sensitivity of the TSCF, where the red line is a linear fit of the dip wavelength shift. (TSCF tapered seven-core fiber, RI refractive index, SLD superluminescent diode, SMF single mode fiber, OSA optical spectrum analyzer) (Note: Error bars were calculated using standard deviation of measurements for three times).

Figure 2a illustrates a schematic diagram of experimental setup. The transmission spectra of several TSCF structures with different *D* values (10, 15, 20, 25, 45, and 60 μm) through the central core are shown in Fig. 2b. Figure 2c shows the transmission curves of the TSCF with tapered diameter *D* of 12 μm when the RI of the solutions varies around a value of ~1.333.

Figure 2d shows the RI sensitivity of the TSCF around an RI value of 1.333. The blue, red, and black curves are the linear fitting of the dip wavelength shift for *D* of 12, 16, and 25 μm, respectively. When *D* = 12 μm, the experimental result (1188.5 nm/RIU) agrees well with the result simulated (1220 nm/RIU) as in Fig. 1g. Because biochemical sensing is typically conducted in aqueous solutions, we measured the temperature sensitivity of the TSCF in water. Figure 2e shows the temperature response, with a sensitivity of −69.7 pm/°C (the Supplementary Material 2 contains a thorough description).

### The functionalization of TSCF biosensor

**Fig. 3:**
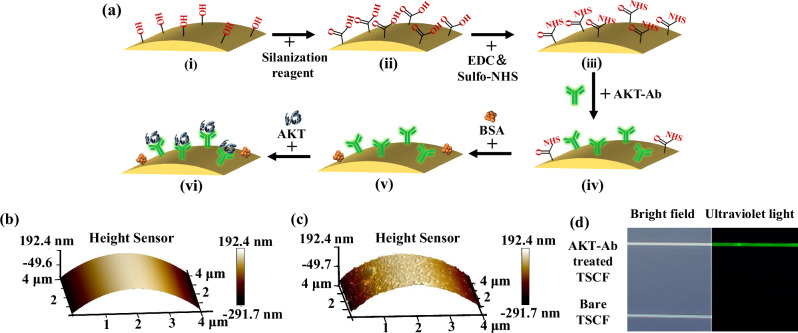
Modification process and effect characterization of the TSCF biosensor surface. **a** Modification process, (**i**) hydroxyl groups created with a KOH solution treatment, (**ii**) carboxyl groups created with a silane reagent treatment, (**iii**) NHS active ester generated with EDC/Sulfo-NHS, (**iv**) AKT-Ab immobilized on the TSCF biosensor surface, (**v**) unbound sites blocked with the BSA, (**vi**) the sensor is ready for specific binding with AKT. **b** AFM image of the TSCF biosensor surface before being modified. **c** AFM image of the TSCF biosensor surface after being modified showing AKT-Ab bound to the TSCF surface. **d** Images of a fluorescent AKT-Ab treated TSCF and a bare TSCF, under both visible and ultraviolet light. (TSCF tapered seven-core fiber, EDC C_8_H_17_N_3_·HCl, Sulfo-NHS C_4_H_4_NNaO_6_S, BSA bovine serum protein, AKT-Ab AKT antibody, AFM atomic force microscope).

If the TSCF biosensor is functionalized with a layer of AKT-Ab, it can be used for AKT sensing. The underlying principle is that when AKT binds to the AKT-Ab layer fixed on the surface of TSCF, the properties of the layer change, specifically the layer’s effective RI and thickness, resulting in a shift in transmission wavelength for the TSCF. Assuming the relationship between wavelength shift and AKT concentration has been calibrated, the biosensor of TSCF functionalized by AKT-Ab can be used to detect the level of AKT. The functionalization procedure is shown graphically in Fig. 3a (i–vi)^21^ (Supplementary Material 3 contains a thorough description). Figure 3b, c show the atomic force microscope (AFM) (Dimension ICON, Bruker, Germany) images of the TSCF surface before and after immobilization of AKT-Ab (corresponding respectively to Fig. 3a (i) and Fig. 3a (v)). In order to provide confirmation that AKT-Ab can be successfully immobilized onto the TSCF surface, a TSCF modified with a fluorescent AKT-Ab (product code: CL488- 60203, Proteintech) was prepared. Figure 3d shows a microscope image of the fluorescent AKT-Ab treated fiber under both visible and ultraviolet light, with a second bare TSCF fiber for comparison. While no difference can be seen between the two fibers under visible light, under ultraviolet light, the fluorescence from the AKT-Ab treated fiber is clear, confirming that AKT-Ab can be successfully immobilized onto the TSCF surface using the process depicted in Fig. 3a.

### Reporting summary

Further information on research design is available in the Nature Portfolio Reporting Summary linked to this article.

## Results and discussion

### The detection of AKT in solution

**Fig. 4:**
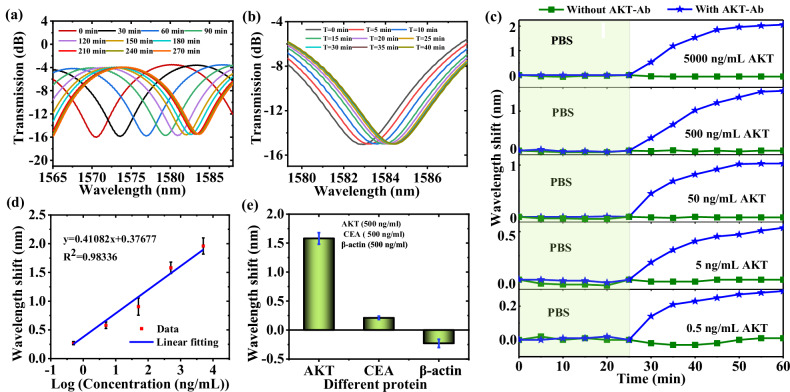
The sensing performance of the TSCF biosensor to AKT protein. **a** The transmission spectra as functions of time for AKT-Ab immobilization on TSCF surface. **b** The transmission spectra as a function of time in a 500 ng/mL AKT solution. **c** The wavelength shifts vs time for different concentrations of the AKT solution. **d** Wavelength shift vs concentration of AKT. **e** Specificity results for the TSCF biosensor. (TSCF tapered seven-core fiber, AKT-Ab AKT antibody) (Note: Error bars were calculated using standard deviation of measurements for three times).

The concentration of primary antibodies used for the functionalization of TSCF biosensors in the subsequent experiments is 20 μg/mL (The recommended dilution ratio for Immunoprecipitation). Figure 4a shows the spectral responses every 30 min of the biosensor during the AKT-Ab (product code: 10763-T16, Sino Biological) functionalization process over 4 h (The spectrum between 1530 nm and 1600 nm is shown in Fig. S3 in the Supplementary Material). As shown in Fig. 4a, the wavelength was red-shifted as time increased, and the total wavelength shift was 13.17 nm. In the first 2 h, the transmission spectrum changed rapidly and then slowed down after 3.5 h, indicating that the AKT-Ab immobilizing process mainly takes place in the first 3.5 h.

Prior to introducing AKT solution, the functionalized TSCF biosensor was immersed into PBS buffer for a (pH = 7.4) period of 25 min as a test of stability. If the wavelength shift of the biosensor in PBS did not exceed 0.05 nm, then it was immersed into AKT protein solution for about 35 min, while at the same time its spectral response was recorded every 5 min. In our experiments, five different concentrations (0. 5, 5, 50, 500, and 5000 ng/mL) of AKT protein (product code: 10763-H08B, Sino Biological) solution were used for sensor tests. Figure 4b shows the spectral response versus time in a 500 ng/mL AKT protein solution (The spectrum between 1530 nm and 1600 nm is shown in Fig. S4 in the Supplementary Material). As seen from Fig. 4b the wavelength undergoes a red-shift, in the first 20 min, the transmission spectrum changed rapidly but the rate of change slows down after 30 min, indicating that a large proportion of the specific capture between AKT-Ab and AKT protein takes place primarily in the first 20 min, after which binding is saturated. The blue curves in Fig. 4c show the measured wavelength shift of the TSCF biosensor over time (including the stability measurement in the PBS solution) for the five AKT solutions. In addition, the effect of non-specific drift of the TSCF biosensor was studied by using a blank TSCF sensor, made using the same fabrication and functionalization parameters as other sensors, but without the step involving immobilization of an antibody. As shown in Fig. 4c, the wavelength shift induced by non-specific binding for the blank TSCF is very low, less than 0.05 nm. The reason for the near-zero non-specific drift is that protein molecules possess a hydration shell and surface charge, which allows the protein solution to exist in a stable form as a colloidal solution without deposition. The experimental results show that an AKT concentration low as 0.5 ng/mL can be reliably detected. Reproducibility of the TSCF biosensor for every sample was evaluated using three TSCFs with the same structure and dimensions. Each biosensor functionalized is used only once. In total, fifteen TSCF biosensors were used to detect AKT solutions with concentrations of 5000, 500, 50, 5, and 0.5 ng/mL, and the mean peak wavelength shifts of the three repeated experiments were 2.06, 1.51, 1.03, 0.54, and 0.29 nm, respectively. The linear fitting results after calculating error bars were shown in Fig. 4d. The experimental results show that the wavelength shift *y* is linearly changed as the logarithm of the AKT concentration *x* varies and can thus be represented by the function:

1 $$y=0.41082{{{\mathrm{lg}}}}[x({{{\rm{ng}}}}/{{{\rm{mL}}}})]+0.38$$

The value of *R*^2^ is 0.98336, indicating a good linear relationship between wavelength shift *y* and logarithm of the AKT concentration *x*.

The standard deviations are 0.02828, 0.05657, 0.14849, 0.09899, 0.014142 for the five concentrations measured in Fig. 4d, and the corresponding coefficient of variation are 10.5%, 9.8%, 16.4%, 6.3% and 7.2%, respectively, which is indicative of good reproducibility.

According to the limit-of-detection (LOD) Eq. (1)^54^

2 $${{{\rm{LOD}}}}=3.3\times 2.303{S}_{y}x/[{{{\rm{d}}}}y/{{{\rm{d}}}}({{\mathrm{lg}}}x)]$$

3 $${S}_{y}=\sqrt{\frac{{\sum ({y}_{i}-\bar{y})}^{2}}{{FD}}}$$

where *S*_*y*_ denotes the standard deviation of responses, *FD* is the degree of freedom, which is 3. In Fig. 4d, x is 0.5 ng/mL, d*y* /d (lg *x*) is 0.41082, *S*_*y*_ is calculated to be 0.02828 at the concentration 0.5 ng/mL, therefore the LOD of this TSCF biosensor is calculated to be 0.26 ng/mL.

Furthermore, the specificity of the TSCF biosensor was also investigated. Nine identical TSCF biosensors were fabricated with AKT-Ab as above and were used to detect different samples: 500 ng/mL AKT solution, 500 ng/mL CEA solution, and 500 ng/mL *β*-actin protein solution. Each type of solution was tested three times. Figure 4e shows the test results with error bars to provide an understanding into the repeatability tests. As seen from Fig. 4e, with the same concentration, the corresponding wavelength variation of AKT solution is much larger than that of CEA solution and *β*-actin protein. The corresponding average wavelength shifts are 1.58, 0.21, and −0.23 nm, respectively, confirming the excellent specificity and repeatability of the TSCF biosensor. It is noted that for the *β*-actin solution, there is blueshift for the spectral response of the sensor. This is possibly because the solvent for the *β*-actin protein (Solarbio, P02410) contains sodium dodecyl sulfate - polyacrylamide gel electrophoresis (SDS-PAGE), which can separate proteins. The negative signal in *β*-actin could be due to the presence of SDS in the protein solution, which remove the antibodies from the sensor surface.

### The detection of AKT in LoVo cell protein

AKT has been reported as having an ability to promote cell survival by inhibiting apoptosis, and its activation is associated with the phosphorylation of Thr308 and Ser473 residues (referred to as p-AKT)^7^. The changes in AKT and its activated component p-AKT in cancer cells reflect the changes of cell activity. In general, p-AKT levels in cancer cells are higher than those in normal cells. A decrease in p-AKT level indicates that the proliferation of cancer cells is inhibited^4,10^. Therefore, in order to test the reliability of the TSCF sensing method in an actual medical application, the proposed TSCF biosensor was used to detect the changes in AKT levels and its activating component p-AKT in cellular proteins as a means to investigate the impact of chrysin on LoVo cells. The cellular proteins were also detected by traditional western blotting method, and the results were compared with those obtained by the proposed biosensor.

**Fig. 5:**
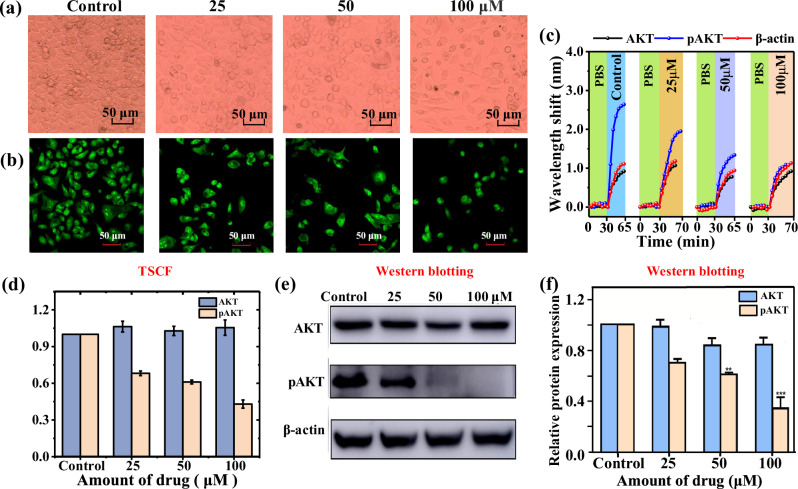
Characterization of LoVo cells and detection of AKT in LoVo cell protein by TSCF biosensor and WB. **a** The morphology micrographs of LoVo cells after chrysin treatment for 24 h obtained by a phase contrast microscope (×100). **b** The morphology of LoVo cell nuclei after chrysin treatment for 24 h by staining with acridine orange. **c** Wavelength shift (black line for AKT, blue line for p-AKT, and red line for *β*-actin) versus time in different LoVo cell protein solutions with treatment of different concentrations of chrysin (0, 25, 50, 100 μmol/L). **d** Normalized wavelength shifts detected by TSCF biosensor of AKT and p-AKT in LoVo cell protein solutions with treatment of different concentrations of chrysin (0, 25, 50, and 100 μmol/L). **e** Expression of AKT and p-AKT after chrysin (0, 25, 50 and 100 μmol/L) treatment detected by WB at 24 h. **f** Relative representation quantization map of AKT and p-AKT in LoVo cells using WB. (TSCF tapered seven-core fiber, WB western blotting) (Note: Error bars were calculated using standard deviation of measurements for three times).

In the experiments, LoVo cells were divided into four groups and cultured in a 6-well plate for 24 h, consisting of a control group and chrysin groups (25, 50, and 100 μmol/L). Cells in the control group were cultured normally. Cells in the chrysin groups were treated with different concentrations of chrysin (25, 50, and 100 μmol/L). Cell morphology after chrysin treatment for 24 h taken by a 100X optical microscope is shown in Fig. 5a, and the morphological changes of nuclei taken by a confocal laser scanning microscope (Olympus FV1200, Japan) are shown in Fig. 5b after acridine orange staining. Figure 5a, b show that LoVo cells were markedly inhibited by chrysin and the inhibition effect varied with doses used. (Supplementary Material 5 contains a thorough description of the culture of cells).

In order to study the quantitative change in the AKT level, the total protein content of the cells was extracted first (Supplementary Material 6 contains a thorough description of the extraction of LoVo cell protein). Each total cellular protein was then diluted to the same concentration of 10 μg/mL. To correct the errors in the protein quantification and diluting process, *β*-actin protein was applied as a reference to ensure the accuracy of the experimental results, since its expression in each tissue cell is relatively constant^51,55^. Each cell protein solution was tested by three TSCF biosensors with the same fabrication parameters and functionalized with one of three different antibodies, all diluted at a 1:50 rate (about 20 μg/mL): AKT-Ab (product code: 9272S, Cell Signaling Technology, USA), p-AKT-Ab (product code: 9271S, Cell Signaling Technology, USA), and *β*-actin antibody (product code:3700S, Cell Signaling Technology, USA). The corresponding wavelength shifts vs time for AKT, p-AKT, and *β*-actin for the four samples are shown is Fig. 5c. For the purpose of comparison, they have been time-biased and placed in a graph. In particular, Fig. 5c shows that the wavelength dip has a red shift in the protein solution extracted from LoVo cell, where the wavelength shifts of *β*-actin in the four groups are similar, indicating that the protein concentration of the four samples tested is almost equal. Figure 5c also shows that the wavelength shift of p-AKT in the control group is larger than that of AKT, indicating that the concentration of p-AKT is greater than that of AKT in LoVo cells that were not treated with chrysin. The corresponding wavelength shift of p-AKT in the treated group decreased noticeably with the increase of chrysin concentration, while corresponding wavelength shift of AKT did not change substantially.

To compensate for the difference caused by the protein sample concentration difference, the wavelength shifts of AKT and p-AKT were normalized by dividing them by the wavelength shift of *β*-actin. For convenience in the comparison, the results were normalized with the control experiment serving as a reference, just like the WB method does. Each protein sample (AKT, p-AKT, and *β*-actin) was detected 3 times and each measurement was conducted using an individual functionalized TSCF biosensor. The average wavelength shifts were normalized as 1, 1.06, 1.03, and 1.06 nm associated with the AKT, and 1, 0.68, 0.61, and 0.43 nm associated with the p-AKT in LoVo cell proteins. A column chart with error bars were then prepared as shown in Fig. 5d. As seen from Fig. 5d, with an increase in the drug dosage of chrysin, the measured concentration of AKT did not change much, but the concentration of p-AKT decreased considerably, compared to that of control group. The results indicate that chrysin has limited impact on the AKT level, but does decrease the p-AKT level, suggesting that one reason for inhibitory effect of chrysin on LoVo cell proliferation is the dephosphorylation of AKT, rather than a decrease in the total AKT levels.

AKT and p-AKT within cell proteins were also tested using conventional WB, with *β*-actin acted as the internal refs. ^51,55^ (The main steps of WB are shown in Supplementary Material 7). The detection result strips are shown in Fig. 5e. Their intensities were processed with ImageJ software. The intensity of *β*-actin served as a reference to quantify the relative expression levels of AKT and p-AKT. Then the value of the control group was set to 1 for normalization. A column chart and statistical analysis were then performed as shown in Fig. 5f. Compared with the control group, the phosphorylation level of AKT decreased significantly after treatment with chrysin (25, 50, and 100 μmol/L), but the change of total AKT level was small, indicating that chrysin can effectively inhibit AKT activation. The results measured by the TSCF biosensor are consistent with these measured by WB.

## Conclusions

In this work, a novel TSCF biosensor based on supermodal interferometry is proposed. Experimental results demonstrate that the TSCF biosensor developed in this paper has an underlying RI sensitivity of 1188.5 nm/RIU around an RI of 1.33 and a temperature sensitivity of −69.7 pm/°C. The developed TSCF structure has been functionalized with AKT-Ab with concentration of 20 μg/mL for AKT protein detection, in order to demonstrate its biosensing capability. As a practical example of the application of the sensor, the sensor was then used to study the anti-cancer effect and mechanism of chrysin on LoVo cells. The results show that the TSCF biosensor can verify that chrysin has limited impact on the AKT level, but decreased the p-AKT level noticeably, suggesting that one mechanism for the inhibitory effect of chrysin on LoVo cell proliferation is the AKT dephosphorylation, without a decrease in the total AKT levels. The results compare well with the equivalent results acquired using the WB process. A Table comparing the key attributes for the sensor reported here with other reported methods is included in the Supplementary material.

Compared to the WB technique, the microfiber biosensor based detection sensor described here has several advantages which provide a compelling rationale for the use of this microfiber biosensor: (1) There is no need to isolate cellular proteins, which simplifies the initial steps in the detection process; (2) there is no need to use secondary antibodies, which not only simplifies the procedure but also reduces cross-reactions associated with other reagents; (3) the microfiber biosensor allow for very short detection times of about 30 min, which is substantially faster than that for the WB technique. In addition, the risk of protein degradation and denaturation is reduced due to the shortened detection time. We believe that the proposed sensor is a simple, rapid, and reliable alternative method for quantitative detection of intracellular marker protein concentration, which will greatly improve the research and treatment for cancer, stroke, and diabetes. Furthermore, the developed TSCF biosensor can be used as a platform in a variety of other fields that can be diagnosed through the detection of specific antibodies or antigens, such as early diagnosis of cardiovascular diseases, infectious diseases, and allergic diseases.

## Supplementary information

Supplementary Information

Reporting Summary

## Data Availability

All data needed to evaluate the conclusions are present in the paper and Supplementary Material. Additional data related to this paper may be requested from the corresponding authors upon reasonable request.
