# Supplementary material for: Fast detection of protein kinase B in chrysin treated colorectal cancer cells using a novel multicore microfiber biosensor
Source: Commun Eng. 2024 Dec 26;3:185. doi: 10.1038/s44172-024-00332-y (PMC11671600; doi:10.1038/s44172-024-00332-y)
Supplement: Supplementary file 1 — Supplementary Information [file 44172_2024_332_MOESM1_ESM.pdf]

Fast detection of protein kinase B in chrysin treated colorectal cancer cells using a novel multicore microfiber biosensor

Zhen Tian[1, 2,†], Hongzhuan Xuan[3,†], Yicun Yao[2], Shengyu Hao[2], Zhichao Zhang[1], Bingyuan Zhang[2], Jingao Zhang[1], Liqiang Zhang[2], Xinzhu Sang[1], Jinhui Yuan[1]*, Gerald Farrell[4], and Qiang Wu[1,5,6,]*

[1]State Key Laboratory of Information Photonics and Optical Communications, Beijing University of Posts and Telecommunications, Beijing 100876, China.

[2]School of Physics Sciences and Information Technology, Liaocheng University, Liaocheng 252059, China.

[3]School of Life Science, Liaocheng University, Liaocheng 252059, China.

[4]Photonics Research Centre, School of Electrical and Electronic Engineering, City Campus, Technological University Dublin, Dublin 7, Ireland.

[5]Faculty of Engineering and Environment, Northumbria University, Newcastle Upon Tyne NE1 8ST, United Kingdom.

[6]Key Laboratory of Optoelectronic Information Science and Technology of Jiangxi Province, Nanchang Hangkong University, Nanchang 330063, China.

*E-mail addresses: yuanjinhui81@bupt.edu.cn (J. Yuan), qiang.wu@northumbria.ac.uk (Q. Wu).

†These authors contributed equally to this work.

1. The proposed microfiber biosensor structure and experiment

The SCF used in the study is a commercially available product (SM-7C1500, Fibercore Company), whose cross-section structure is shown in Figure 1(a), where the distribution of the seven cores is symmetrical - a central core (core 1) and six satellite cores (cores 2-7). The mode field diameter d of each core is 5.7 - 6.5 μm @1550 nm, the diameter of the cladding is 125 μm and the spacing distances (r and δ) between core are all 35 μm, as described in reference (1). Due to the relatively large distance between cores, the cores of an untapered SCF are only weakly-coupled. In this work, the SCF was tapered adiabatically with an optical glass processing system (3SAE Technologies, USA). The optical fiber is meticulously positioned on two movable platforms and securely clamped by two specialized fixtures. During the tapering process, a unidirectional pulling program is utilized, where the optical fiber is precisely heated by three electrodes to achieve a controlled softening. Simultaneously, the platforms move in concert to ensure that the fiber is uniformly elongated in one smooth operation. To prevent any damage to the fiber core that caused by the diffusion of doping

elements, the entire tapering process deliberately excludes the step of flame scanning.

As the SCF was tapered, d, r, and δ were reduced proportionately with D, according to the characteristics of quartz. The smaller the d, the more the evanescent field overflows the core, the smaller the r and δ, the more overlapping the evanescent fields are. When d, r and are reduced to a certain size, energy exchange between the core modes will take place to form seven supermodes. Figure 1 (b) shows the schematic diagram of the tapered SCF (TSCF) structure, where L_1 and D are the taper waist length and the diameter, respectively, and L_2 is the transition zone length. The central core 1 acts as the input and output light guides of the TSCF. The profile of the tapered SCF with D as 12 μm is shown in Figure S1, Figure S1(a) is detected by the optical glass processing system, Figure S1(b) is detected by a 1000×CCD. As can be seen from the figure, the diameter D of the cone region is uniform, and the diameter change of one transition region is gentle, while the diameter change of the other transition region has a bump. This is because in order to avoid the damage of fiber core by multiple heating, the unidirectional tapering procedure is used, and the bulge formed in the second transition zone due to the uneven stress.

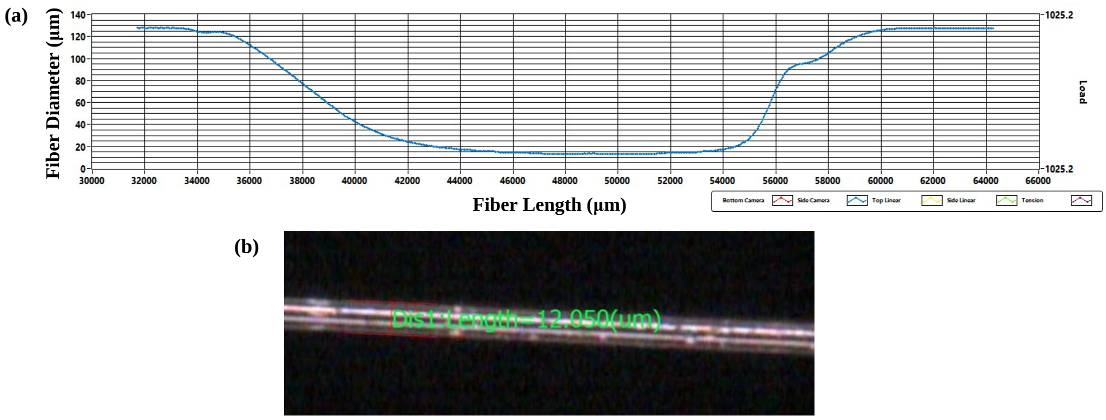

Figure S1. Characterization of the tapered seven-core fiber (a)The profile of the TSCF detected by 3CMS, (b) The diameter of the TSCF detected by 1000×CCD

Using the beam propagation method, the normalized power and propagation profile (blue curve for central core, green curve for one satellite core) along the segment of TSCF at wavelength 1550 nm were simulated, as shown in Figure 1(c). In the simulation, D is chosen as 12 μm, L_1 is chosen as 10 mm, and L_2 is chosen as 8 mm, these values are chosen based on an approximate estimate of the likely values to be used in later experimental work. Figure 1(c) shows that the incident light is strongly confined in the core of the input untapered region. Within the tapered region, due to the reduction of the core-to-core pitch r and δ and diameters of core d along the tapered transition region, the evanescent field of the central core mode extends to overlap the six outer core regions so that the individual cores are no longer independent. As a result, intermodal coupling between the central and outer core mode is taken place so that the light energy will be coupled from the central core to outer cores. Furthermore, there will be an energy exchange among each of the core modes to form supermodes. After the light has passed through the tapered segment, the light field is divided and propagates in all the cores.[1] With the finite element method, the profiles of fundamental and higher-order supermodes of the TSCF were also simulated. Since each core supports only single mode transmission, seven supermodes can be supported[2-4]. Among the multiple supermode fields obtained by the simulation calculation, the first seven supermode fields are selected and shown in Figure 1 (d), while the corresponding Ex component of different supermodes are shown in Figure 1(e). It can be seen that due to the proximity of the seven cores relative to each other and the expansion of the mode field of each core, the individual cores are no longer independent, so that this composite structure forms supermodes of all orders.[5]Due to the differences in the field distributions of the modes, their effective refractive indices are different[6]. When the light is

launched into the structure, different orders of supermodes are excited at the same time. If the central core is used as the input core, since the incident field is axially symmetric, the first two axisymmetric modes will have the highest coupling efficiency and therefore the highest excitation efficiency for the largest overlap integral with the incident field, and they are the components mainly involved in interference 5. As a result, a transmission interference spectrum mainly involving the two modes is formed. In sensing applications, because there is a stronger interaction between the higher-order supermode (the one with a lower effective RI) and the environment, the sensor with higher-order supermode is more sensitive to the external environment change. Therefore, the change of the external environment will cause the change of the phase difference between the two modes, and lead to a spectral shift which in turn enables the sensing function. Figure 1(f) shows the transmission spectra simulated in the RI range from 1.332 to 1.340 when the effective RIs of the core and cladding are set as 1.459 and 1.444 at a wavelength of 1550 nm, respectively (The RI of the diluent PBS buffer used for biosensing is about 1.335). Figure 1(f) shows that as the RI increases the dip wavelength of the TSCF has a monotonic red-shift. The corresponding linearly fitted results between the dip wavelength and RI are shown in Figure 1 (g). As seen from Figure 1(g),an RI sensitivity of 1220 nm/RIU can be achieved within RI range from 1.332 to 1.340.

2.The TSCF sensing system and its sensing perference

Figure 2(a) illustrates a schematic diagram of experimental setup, in which the SLD is a superluminescent diode serve as the light source, which can emit a supercontinuum of light over 1250-1650 nm with low coherence. OSA is an optical spectrum analyzer (YOKOGAWA AQ6370D, Japan), which was used to measure the transmission spectra of the sensor. During the experiments, both ends of the optical fiber sensor are fixed on a U-shape acrylic frame. The fiber sensor section of the U-shape frame is placed to a container (volume 1 mL) with grooves on both sides to allow the connecting fiber of the sensor to go through. The testing liquid was the mixture of different proportions of deionized water and glycerin.

The RI of each test sample was determined using an Abbe refractometer (NAR-3T ATAGO). The transmission spectra of several TSCF structures with different *D* values (10, 15, 20, 25, 45, and 60 μm) through the central core are shown in Figure 2(b). The waist lengths L_1 were ~10mm and transition zone lengths L_2were ~ 8 mm for each structure. It can be seen that the FSR decreases with the decrease of the taper diameter when the length of the taper waist remains constant. Since the RI of the diluents PBS and pure water commonly used in biosensing lie in the range circa 1.33~1.34, we studied the RI sensitivity of the TSCFs near 1.33. Figure 2(c) shows an example of spectra of a TSCF whose *D* is about 12 μm and the surrounding RI is varied around a value of ~1.33. The RI responses of the sensor for different values of *D* (25, 16, and 12 μm) are shown in Figure 2 (d). Both(c) and Figure 2(d) show that, as the surrounding RI increases, the wavelength has a monotonic red-shift, and the RI sensitivities increase from 388.5 to 1188.5 nm/RIU as the tapered diameter *D* decreases from 25 to 12 μm. The main reason that smaller *D* leads to an larger RI sensitivity is that as *D* decreases, a larger proportion of the energy in the evanescent waves reacts with the surrounding liquid, which results in a higher sensitivity. When *D* is 12 μm, the experimental result (1188.5 nm/RIU) agrees well with the result simulated (1220 nm/RIU) as in Figure 1(g). Additionally, for completeness, the RI responses with different RI ranges from 1.3748 to 1.3808 and from 1.4206 to 1.4236 were also investigated, and the RI sensitivities were measured to be 2017.72 and 6780.7 nm/RIU, respectively.

Change the container in Figure 2 to a 500ml square box, the temperature response of the TSCF is studied with the TSCF sample in 400 mL water. The temperature of water is detected by an electronic thermometer placed in adjacent to the TSCF. At first, the water is heated to 70 ℃, then the transmission spectra of the TSCF is recored when the temperature drops every 5 ℃. Figure S2 shows the spectral response of the TSCF when temperature varies from 65 ℃ to 30 ℃. It can be seen that the transmission curves experience blue -shift with the increase of temperature, and the corresponding linear fitting results between the peak wavelength and temperature are shown in Figure 2(e).When temperature varies from 65 to 30 ℃, the temperature sensitivity is -69.7 pm/ ℃. The linear fitting coefficient of determination R^2 is about

0.99968, indicating a good linearity to temperature. The temperature cross-sensitivity for the RI measurement is estimated 5.86×10^{-5} RIU/ ℃ around an RI of 1.33, which is so small that the TSCF can to operate in a stable manner in the presence of relatively small local temperature shifts, without the need for active temperature control of the environment surrounding the sensor.

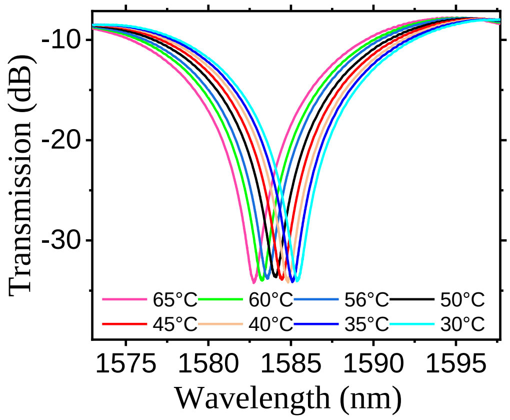

Figure S2. Measured spectral responses at different temperature.

3. The functionalization procedure of TSCF

If the TSCF biosensor is functionalized with a layer of AKT-Ab, it can be used for AKT sensing. The underlying principle is that when AKT binds to the AKT-Ab layer fixed on the surface of TSCF, the properties of the layer change, specifically the layer's effective RI and thickness, resulting in a shift in transmission wavelength for the TCSF. Assuming the relationship between wavelength shift and AKT concentration has been calibrated, the biosensor of TSCF functionalized by AKT-Ab can be used to detect the level of AKT.

The functionalization procedure is shown graphically in Figure 3(a) (i)-(vi). Prior to functionalization, the TSCF biosensor was cleaned with ethanol and then washed three times using deionized water to remove surface impurities. Then the TSCF is functionalized by following the steps (i) to (vi) below[2].

i. Immerse the TSCF biosensor in a 0.1 mol/L standard KOH solution (Aladdin, China) for 1 hour to produce -OH groups on the TSCF surface. Then, wash it three times with deionized water (DI) until the rinse water is neutral (pH=7). Finally, allow it to dry naturally for 0.5 hour.

ii. Immerse the TSCF biosensor in 5% $C_{13}H_{24}O_6Si$ (CAS:93642-68-3, Macklin, China) in ethanol (Aladdin, China) for 4h to produce – COOH groups on the TSCF surface.

iii. Clean the TSCF biosensor one time with ethanol (Aladdin, China), then two times with a pH=6.0 buffer solution (Aladdin, China) and allows to dried naturally for 0.5 hour. Then immerse the biosensor in a mixed solution of $C_8H_{17}N_3$ HCl (0.8 mg/ml) (CAS: 25952-53-8, Aladdin, China) and $C_4H_4NNaO_6S$ (1.2 mg/ml) (CAS: 106627-54-7, Aladdin, China) for one hour in order to functionalize the TSCF surface with NHS active ester groups.

iv. Clean the TSCF biosensor three times with 1×PBS (pH=7.4), then immediately immerse it into an AKT-Ab (Sino Biological, China) solution (diluted with PBS) (Solarbio, China) with a concentration of 20 µg/mL at room temperature for 4 h, then AKT-Ab can be modified on the TSCF biosensor surface.

v. Clean the TSCF biosensor three times with 1×PBS (pH=7.4), and then block it using 1% Albumin Bovine (Sigma, USA) solution for 1 hour at room temperature.

vi. The functionalized biosensor is now ready for specific binding with AKT.

Figure S3 is the wavelength shifts of the biosensor during the functionalization functionalized with AKT-Ab (product code:10763-T16, Sino Biological) with concentration of 20 µg/mL over 4 hours. The part in the red dot line is the part shown in the Figure 4(a) of the paper.

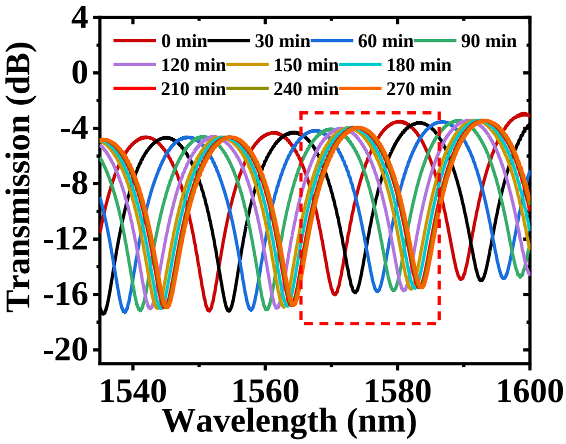

Figure S3. The transmission spectra as functions of time in AKT Anti-body solution

4. Using the TSCF as a biosensor for the AKT detection

Figure S4 shows the transmission spectral responses of the functionalized TSCF biosensor in the range of 1530 nm and 1600 nm after immersion into the AKT solution versus time. The part in the red dot line is the transmission spectra in the Figure 4(b) of the paper.

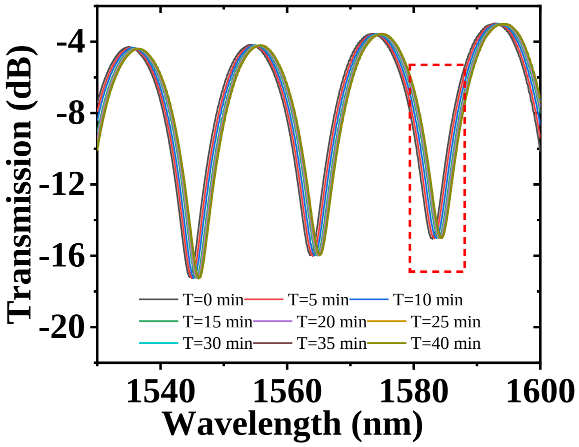

Figure S4. The transmission spectra as functions of time in 500 ng/mL of AKT solution.

After being functionalized, a stability test has been carried out by immersing the fabricated TSCF fiber sensor into PBS buffer which is also used for diluting AKT in the bio-sensing tests at a later stage. The result is shown in Figure S5. As shown in Figure S5, three rounds of stability tests have been carried out, and the TSCF sensor has very good stability with wavelength variations of ± 0.05 nm over 30 minutes.

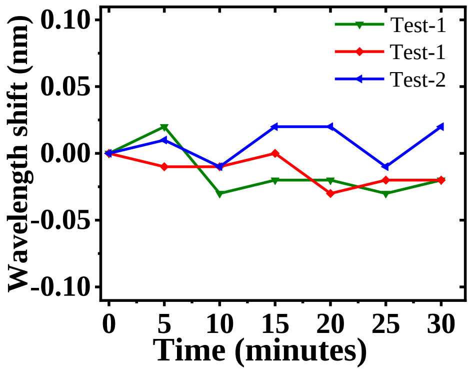

Figure S5. The transmission spectra as functions of time in 500 ng/mL of AKT solution

5. Cell culture

The human colorectal cancer LoVo cell was a gift from the Jining Medical College (China). Chysin (480-40-0) was purchased from Chengdu Mansite Biotechnology CO., LTD. The human colorectal cancer LoVo cell culture and cell protein preparation followed the same method as previously described in reference 6. Human LoVo cells were cultured in culture solution and were placed in incubators (37 ◦C, 5% CO_2, 95% saturated humidity). When the confluence reached a confluence of 75% to 95%, the cells were collected for subsequent experiments.

Human LoVo cancer cells were divided into four groups, consisting of a control and three chrysin (25, 50, and 100 μmol/L) groups. They were cultured partially in a 6-well plate and partially in confocal dishes for 24 h. The control group cells were cultured normally, while the chrysin group cells were cultured with different concentrations of chrysin (25, 50, and 100 μmol/L). Cell morphology micrographs were obtained using a phase contrast microscope at 24 hour (×100). And the morphological changes of nuclei were taken by a confocal laser scanning microscope (Olympus FV1200, Japan) after acridine orange staining.

6.Extraction of human LoVo cell protein

After the cells were cultured for 24 h, they were cleaned twice with ice-cold1×PBS, and then lysed on ice for 30 minutes by adding appropriate amount of RIPA cell lysate (containing 1% protease inhibitor and 1% phosphatase inhibitor). The lysate was collected and hence centrifuged at 13000 rpm at 4°C for 10 minutes. Then the supernatant was collected and its protein concentration was evaluated with an bicinchoninic acid protein assay kit (Beyotime, Jiangsu, China).

7.Western blotting analysis

The main steps of western blotting analysis are shown in Figure S6. Western blotting was performed with 20 μg of Human LoVo cell protein electrophoresed by10% sodium dodecyl sulfate-PAGE. Then the protein was transferred onto PVDF membranes. Hence the membranes were blocked with 5% skim milk at room temperature for 1 hour, and incubated with the primary antibody (1:1000) at 4 ℃ overnight. The following primary antibodies were used: AKT antibody (product code: 9272S, Cell Signaling Technology, USA), p-AKT antibody (product code: 9271S, Cell Signaling Technology, USA), and *β*-actin antibody (product code:3700S, Cell Signaling Technology, USA). After washing the membranes two times for 15 minutes with PBST, the membranes were then incubated with horseradish peroxidase-conjugated secondary antibody (1:5000) at room temperature for 1 hour. Membranes were washed 2 times as above, and then were incubated with enhanced chemiluminescence for immunodetection. Last, the immunoreactive imaging was performed on an Amersham Image600 system (USA).

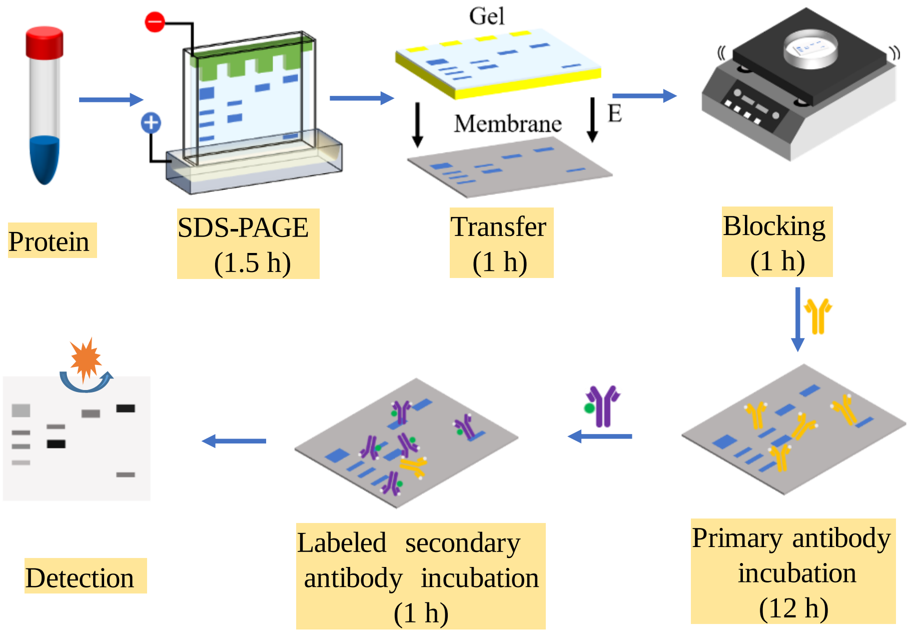

Figure S6. Schematic diagram of Western blotting.

Data availability:

All data needed to evaluate the conclusions are present in the paper and Supplementary material. Additional data related to this paper may be requested from the corresponding authors upon reasonable request.

Supplementary References

1.Tian, Z. et al. Post chemical etching of tapered seven-core fiber sensor for enhanced figure of merit. *Opt. Lett*. **47**, 4672–4675 (2022).

2. Qiu, S. et al. Ultrahigh-sensitivity label-free single mode-tapered multimode-single mode fiber U-shaped biosensor for Staphylococcus aureus detection. *Sens. Actuators B Chem*. **375**, 132927 (2023).

3.Xia, C., Bai, N., Ozdur, I., Zhou, X., & Li, G. Supermodes for optical transmission. *Opt Express*. **19,** 16653-16664 (2011).

4. Xia, C. et al. Supermodes in coupled multi-core waveguide structures. *IEEE J Sel Top Quantum Electron* **22**, 196-207 (2016).

5. Jollivet, C. et al. Mode-resolved gain analysis and lasing in multi-supermode multi-core fiber laser. *Opt Express* **22**, 30377-30386 (2014).

6. Hayashi, Y. et al. Detection limit estimated from slope of calibration curve: an application to competitive ELISA. *Anal. Sci.* **21**, 167–169 (2005).

Competing interests

The authors declare no competing interests.

Acknowledgments

We thank Shuchao Qin for his help in testing the fiber surface with AFM (Dimension ICON, Bruker, Germany). This work was jointly supported by National Natural Science Foundation of China (NSFC) (Granted No. 62175097, 62065013 and 62275015); Royal Society International Exchanges 2022 Round 1(IES\R1\221008); Taishan Scholar Foundation of Shandong Province (NO. tsqn202211172)
