# Supplementary material for: Fast detection of protein kinase B in chrysin treated colorectal cancer cells using a novel multicore microfiber biosensor
Source: Commun Eng. 2024 Dec 26;3:185. doi: 10.1038/s44172-024-00332-y (PMC11671600; doi:10.1038/s44172-024-00332-y)
Supplement: Supplementary file 2 — Reporting Summary [file 44172_2024_332_MOESM2_ESM.pdf]

nature portfolio

Corresponding author(s): Qiang Wu

Last updated by author(s): Dec 9, 2024

Reporting Summary

Nature Portfolio wishes to improve the reproducibility of the work that we publish. This form provides structure for consistency and transparency in reporting. For further information on Nature Portfolio policies, see our Editorial Policies and the Editorial Policy Checklist.

Statistics

For all statistical analyses, confirm that the following items are present in the figure legend, table legend, main text, or Methods section.

n/a	Confirmed	
☐	☒	The exact sample size (n) for each experimental group/condition, given as a discrete number and unit of measurement
☐	☒	A statement on whether measurements were taken from distinct samples or whether the same sample was measured repeatedly
☐	☒	The statistical test(s) used AND whether they are one- or two-sided<br>*Only common tests should be described solely by name; describe more complex techniques in the Methods section.*
☐	☒	A description of all covariates tested
☐	☒	A description of any assumptions or corrections, such as tests of normality and adjustment for multiple comparisons
☐	☒	A full description of the statistical parameters including central tendency (e.g. means) or other basic estimates (e.g. regression coefficient) AND variation (e.g. standard deviation) or associated estimates of uncertainty (e.g. confidence intervals)
☐	☒	For null hypothesis testing, the test statistic (e.g. F, t, r) with confidence intervals, effect sizes, degrees of freedom and P value noted<br>*Give P values as exact values whenever suitable.*
☒	☐	For Bayesian analysis, information on the choice of priors and Markov chain Monte Carlo settings
☒	☐	For hierarchical and complex designs, identification of the appropriate level for tests and full reporting of outcomes
☒	☐	Estimates of effect sizes (e.g. Cohen's d, Pearson's r), indicating how they were calculated

Our web collection on statistics for biologists contains articles on many of the points above.

Software and code

Policy information about availability of computer code

Data collection	Microsoft 365
Data analysis	Microsoft 365

For manuscripts utilizing custom algorithms or software that are central to the research but not yet described in published literature, software must be made available to editors and reviewers. We strongly encourage code deposition in a community repository (e.g. GitHub). See the Nature Portfolio guidelines for submitting code & software for further information.

Data

Policy information about availability of data

All manuscripts must include a data availability statement. This statement should provide the following information, where applicable:

- Accession codes, unique identifiers, or web links for publicly available datasets
- A description of any restrictions on data availability
- For clinical datasets or third party data, please ensure that the statement adheres to our policy

All data needed to evaluate the conclusions are present in the paper and Supplementary Material. Additional data related to this paper may be requested from the corresponding authors upon reasonable request.

Human research participants

Policy information about studies involving human research participants and Sex and Gender in Research.

Reporting on sex and gender	No
Population characteristics	No
Recruitment	No
Ethics oversight	No

Note that full information on the approval of the study protocol must also be provided in the manuscript.

Field-specific reporting

Please select the one below that is the best fit for your research. If you are not sure, read the appropriate sections before making your selection.

☒ Life sciences ☐ Behavioural & social sciences ☐ Ecological, evolutionary & environmental sciences

For a reference copy of the document with all sections, see nature.com/documents/nr-reporting-summary-flat.pdf

Life sciences study design

All studies must disclose on these points even when the disclosure is negative.

Sample size	The cells were divided into 4 groups: control group and 3 different dose groups. Three independent experiments were performed on each cell group.
Data exclusions	No
Replication	Repeat at least 3 times.
Randomization	Sample random allocation.
Blinding	Yes

Reporting for specific materials, systems and methods

We require information from authors about some types of materials, experimental systems and methods used in many studies. Here, indicate whether each material, system or method listed is relevant to your study. If you are not sure if a list item applies to your research, read the appropriate section before selecting a response.

Materials & experimental systems

n/a	Involved in the study	
☐	☒	Antibodies
☐	☒	Eukaryotic cell lines
☒	☐	Palaeontology and archaeology
☒	☐	Animals and other organisms
☒	☐	Clinical data
☒	☐	Dual use research of concern

Methods

n/a	Involved in the study	
☒	☐	ChIP-seq
☒	☐	Flow cytometry
☒	☐	MRI-based neuroimaging

Antibodies

Antibodies used	AKT Antibody, p-AKT Antibody, β-Actin Antibody
Validation	AKT Antibody: Rabbit (Lot: 9272S), Cell Signaling Technology; p-AKT Antibody: Rabbit (Lot: 9271S), Cell Signaling Technology; β-Actin Antibody: Mouse IgG2b (Lot: 3700S), Cell Signaling Technology. They are all available on the website: https://www.cellsignal.com/<br>AKT Antibody: Rabbit (Lot: 10763-T16), Sino Biological. It is available on the website: https://cn.sinobiological.com/antibodies/

akt1-10763-t16. fluorescent AKT Antibody: Mouse (Lot: CL488-60203), Proteintech, It is available on the website: https://www.ptgcn.com/products/AKT-Antibody-CL488-60203.htm.

Eukaryotic cell lines

Policy information about cell lines and Sex and Gender in Research

Cell line source(s)	The human colorectal cancer LoVo cell was a gift from the Jining Medical College (China), and it was purchased from the American Type Culture Collection (Manassas, VA, USA).
Authentication	It has been genotyped and verified by experiments.
Mycoplasma contamination	No mycoplasma had been tested and no mycoplasma has been found in the ongoing experiments.
Commonly misidentified lines (See ICLAC register)	There is no misidentified lines in this experiment.
